# Supplementary material for: Probing the subtle differences between promethium and curium
Source: Nat Commun. 2025 Apr 4;16:3220. doi: 10.1038/s41467-025-58209-3 (PMC11971438; doi:10.1038/s41467-025-58209-3)
Supplement: Supplementary file 1 — Supplementary Information [file 41467_2025_58209_MOESM1_ESM.pdf]

Supplementary Information
Probing the Subtle Differences between Promethium and Curium

Trenton Vogt[1,2]†, Megan Simms[3]†, Connor Parker[3], April Miller[3], Laetitia Delmau[3], Richard Mayes[4], Samantha Cary[3], Alyssa Gaiser[1,2], Cristian Celis-Barros[3]*, Frankie White[3]*

Corresponding author(s): celisbarroca@ornl.gov; whitefd@ornl.gov

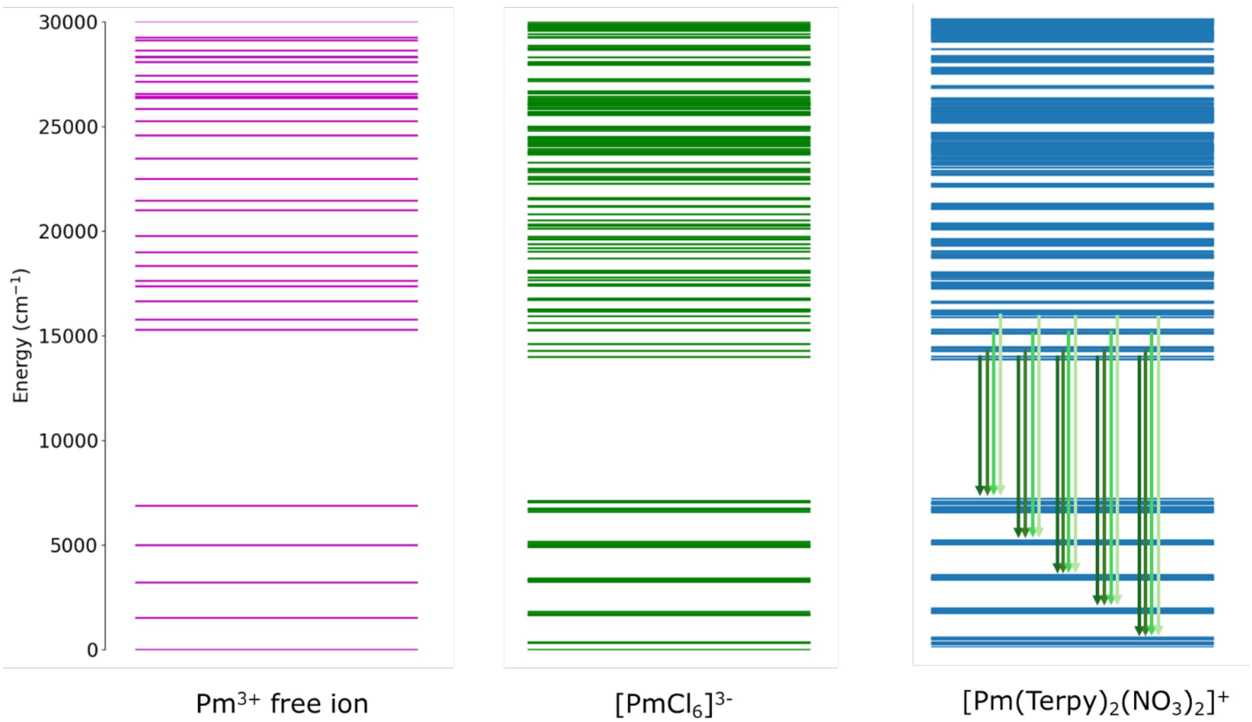

Fig. S1.

Comparison of the calculated multiplet structure of the Pm^{3+} free ion, Pm^{3+} hexachloride, and 2:1 Terpy complex. The calculations show that the splitting of the ^5I_J manifold is similarly split for the chloride and Terpy complexes. The main difference between the free ion and molecular complexes is the stabilization of the $^5F_1$ multiplet by ~ 1,000 cm^{-1}. The expected similar emission bands are present in the molecular complexes.

Table S1.

**Computed structural geometry errors of the NdTerpy, PmTerpy with and without Sm, SmTerpy, and CmTerpy. The daughter isotope of ^{147}Pm is ^{147}Sm and was checked to make sure if up to 20% ingrowth of ^{147}Sm would significantly affect the ^{147}Pm crystal structure. Errors**		**Nd**	**Pm (w/Sm)**	**Pm(no Sm)**	**Sm**	**Cm**
**1:1 complex**	**N**	1.5%	0.3%	0.2%	-0.6%	0.3%
	**O**	-0.3%	-0.5%	-0.8%	-0.5%	-0.1%
**1:2 complex**	**N**	1.7%	-1.3%	-1.4%	0.8%	-0.7%
	**O**	3.7%	0.1%	0.0%	5.4%	0.3%

Table S2.

X-ray crystallographic data collection and refinement parameters for novel $[M(Terpy)(NO_3)_2][M(Terpy)(NO_3)_4]\cdot 2MeCN$ compounds discussed in this work.

Compound	$[Ce(Terpy)(NO_3)_2][Ce(Terpy)(NO_3)_4]\cdot$ 2MeCN	$[Pr(Terpy)(NO_3)_2][Pr(Terpy)(NO_3)_4]\cdot$ 2MeCN
CCDC#	2366325	2366329
Empirical formula	$C_{45}H_{30}Ce_2N_{15}O_{18}$ [+ solvent]	$C_{49}H_{39}Pr_2N_{17}O_{18}$
Formula weight	1349.08	1435.79
a (Å)	11.0882 (13)	11.1709 (3)
b (Å)	17.3390 (15)	16.0121 (4)
c (Å)	17.4815(3)	16.2966 (4)
α (°)	84.881 (6)	106.7430 (10)
β (°)	78.600 (6)	94.9950 (10)
γ (°)	85.512 (6)	95.8450 (10)
V (Å^3)	3027.8 (5)	2756.07 (12)
Z	2	2
Crystal system	Triclinic	Triclinic
Space group	*P-1*	*P-1*
ρ_{calc} (g/cm^3)	1.483	1.730
μ (mm^{-1})	1.560	1.836

T (K)	299.40	299.70
2θ range (°)	3.754 to 50.846	4.264 to 62.06
Independent reflections	10745	16986
R_{int}	0.0592	0.0373
Number of parameters	721	893
Max, min peaks (e·Å^{-3})	1.965, 1.120	0.736, 0.621
R1[a]/wR2[b] (all data)	0.0806/0.1873	0.0552/0.0828
R1[a]/wR2[b] (>2σ)	0.0592/0.1687	0.0373/0.0742
Goodness of fit[c]	1.039	1.014
Compound	$[Pm(Terpy)(NO_3)_2][Pm(Terpy)(NO_3)_4]\cdot$ 2MeCN	$Eu(Terpy)(NO_3)_2][Eu(Terpy)(NO_3)_4]\cdot$ 2MeCN
CCDC#	2366328	2366327
Empirical formula	$C_{49}H_{39}Pm_2N_{17}O_{18}$	$C_{49}H_{39}Eu_2N_{17}O_{18}$
Formula weight	1447.81	1457.91
a (Å)	11.1738 (4)	11.042 (4)
b (Å)	15.9791 (6)	15.985 (5)
c (Å)	16.2596 (6)	16.261 (5)
α (°)	105.8847 (18)	106.206 (17)
β (°)	95.6780 (19)	95.895 (18)
γ (°)	95.970 (2)	96.034 (18)
V (Å^3)	2752.37 (18)	2714.5 (16)
Z	2	2
Crystal system	Triclinic	Triclinic
Space group	*P-1*	*P-1*
ρ_{calc} (g/cm^3)	1.747	1.784
μ (mm^{-1})	2.071	2.380
T (K)	300.40	299.40
2θ range (°)	4.258 to 56.668	4.27 to 57.02
Independent reflections	13384	13317
R_{int}	0.0387	0.0415
Number of parameters	768	777
Max, min peaks (e·Å^{-3})	1.016, 0.735	0.526, 0.835
R1[a]/wR2[b] (all data)	0.0611/0.1034	0.0599/0.1003
R1[a]/wR2[b] (>2σ)	0.0387/0.0911	0.0415/0.0875
Goodness of fit[c]	1.041	1.021
Compound	$[Gd(Terpy)(NO_3)_2][Gd(Terpy)(NO_3)_4]\cdot$ 2MeCN	$[Er(Terpy)(NO_3)_2][Er(Terpy)(NO_3)_4]\cdot$ 2MeCN

CCDC#	2366324	2366323
Empirical formula	$C_{49}H_{39}Gd_2N_{17}O_{18}$	$C_{49}H_{39}Er_2N_{17}O_{18}$
Formula weight	1468.47	1488.49
a (Å)	11.0671 (2)	11.0138 (2)
b (Å)	15.9865 (3)	15.9342 (3)
c (Å)	16.2163 (3)	16.1587 (3)
α (°)	106.0779 (9)	105.4590 (10)
β (°)	95.5905 (10)	96.2170 (10)
γ (°)	95.9480 (9)	95.8240 (10)
V (Å^3)	2718.01 (9)	2756.07 (12)
Z	2	2
Crystal system	Triclinic	Triclinic
Space group	*P-1*	*P-1*
ρ_{calc} (g/cm^3)	1.794	1.836
μ (mm^{-1})	2.510	3.188
T (K)	298.50	299.50
2θ range (°)	4.276 to 64.096	4.276 to 64.094
Independent reflections	18544	17911
R_{int}	0.0430	0.0348
Number of parameters	777	777
Max, min peaks (e·Å^{-3})	0.741, 0.626	0.873, 0.773
R1[a]/wR2[b] (all data)	0.0684/0.0890	0.0527/0.0768
R1[a]/wR2[b] (>2σ)	0.0430/0.0765	0.0348/0.0700
Goodness of fit[c]	1.011	1.030

Compound	[Cm(Terpy)$(NO_3)_2$][Cm(Terpy)$(NO_3)_4$]· 2MeCN
CCDC#	2366326
Empirical formula	$C_{49}H_{39}Cm_2N_{17}O_{18}$
Formula weight	1642.09
a (Å)	11.1057 (2)
b (Å)	15.9836 (3)
c (Å)	16.2475 (3)
α (°)	106.3050 (10)
β (°)	95.1700 (10)
γ (°)	95.9550 (10)
V (Å^3)	2731.59 (9)
Z	2
Crystal system	Triclinic
Space group	*P-1*
ρ_{calc} (g/cm^3)	1.996
μ (mm^{-1})	3.054

T (K)	301.50
2θ range (°)	4.278 to 66.338
Independent reflections	19946
R_{int}	0.0373
Number of parameters	772
Max, min peaks (e·Å^{-3})	1.663, 1.835
R1^a/wR2^b (all data)	0.0534/0.0930
R1^a/wR2^b (>2σ)	0.0373/0.0848
Goodness of fit^c	1.040

^{a} $R_1 = \Sigma||Fo| - |Fc||/\Sigma|Fo|$ for $I > 2\sigma$. ^{b} $wR_2 = \{\Sigma[w(F_o^2 - F_c^2)^2]/\Sigma[w(F_o^2)^2]\}^{1/2}$ for $I > 2\sigma$. ^{c} $GoF = \{\Sigma[w(F_o^2 - F_c^2)^2]/(n - p)\}^{1/2}$, where n is the number of data and p is the number of refined parameters.

Many of the structures possess one or two B-alerts which are all the same. The primary B-alert is the 'plat242_ALERT_2_B' is on the monodentate nitrogen which is bound to oxygens with large thermal ellipsoid parameters, thus presenting a problem with the N-O bond distances. No attempts were made to order or split the O atoms in these structures. The second alert, 'plat913_ALERT_3_B is associated with missing strong reflections. Poor reflections were omitted from the crystal data refinement with a mean I/σ < 3. The structures are isostructural and confirmed not to be in the orthorhombic space group, which would also cause this alert. The reflections may also be affected by the beam stop.

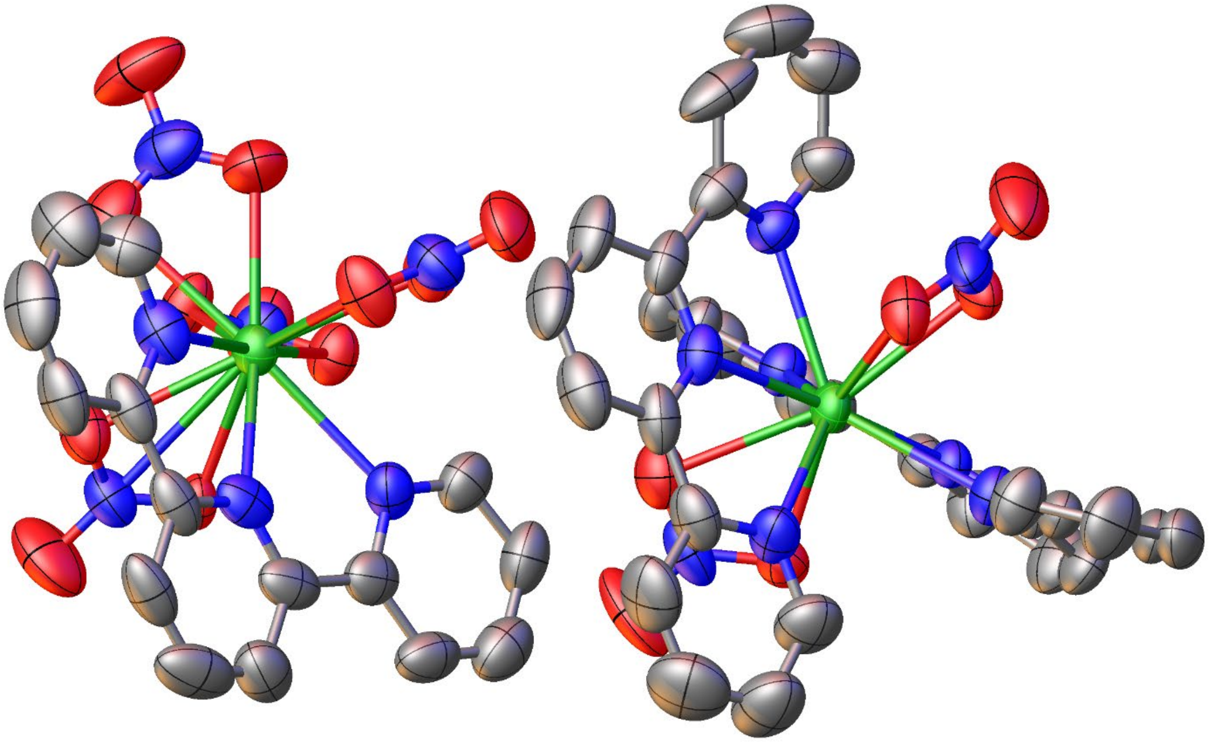

Figure S2. Crystal Structure of CeTerpy (CCDC# 2366325) shown at the 50% probability level. Hydrogens have been omitted for clarity.

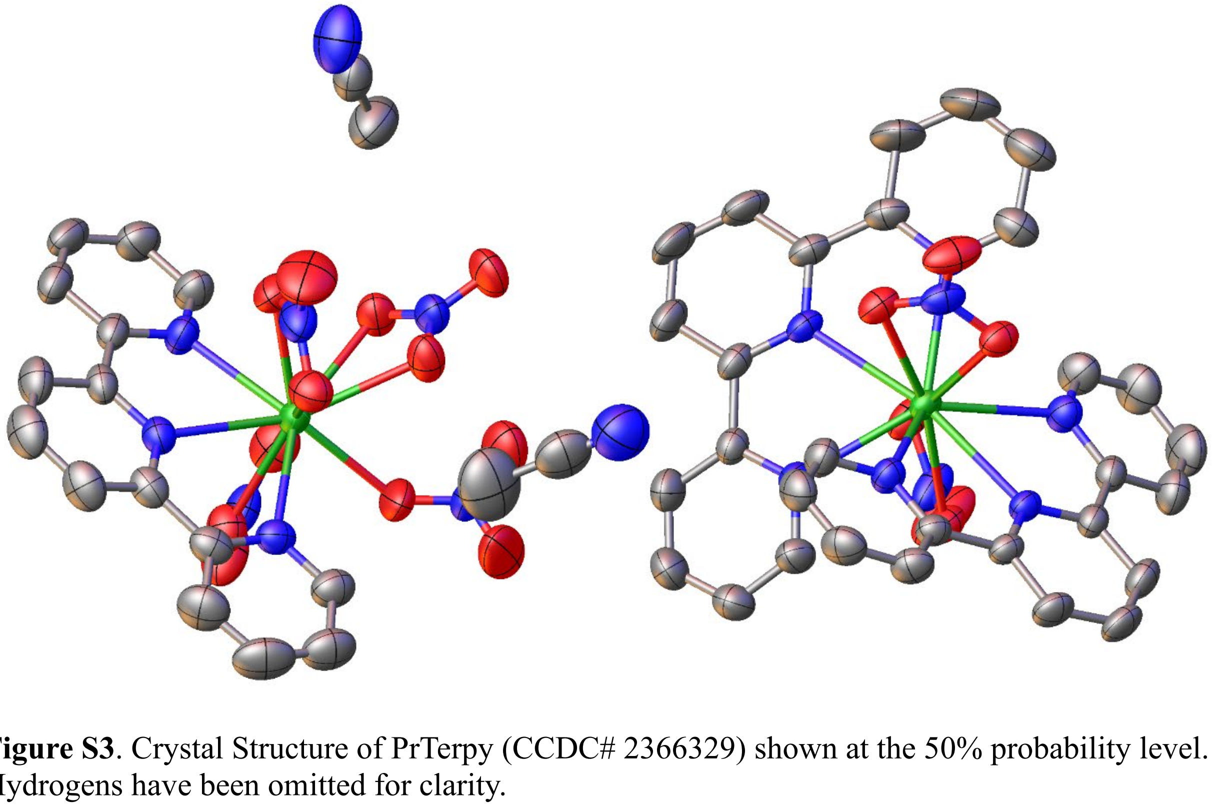

Figure S3. Crystal Structure of PrTerpy (CCDC# 2366329) shown at the 50% probability level. Hydrogens have been omitted for clarity.

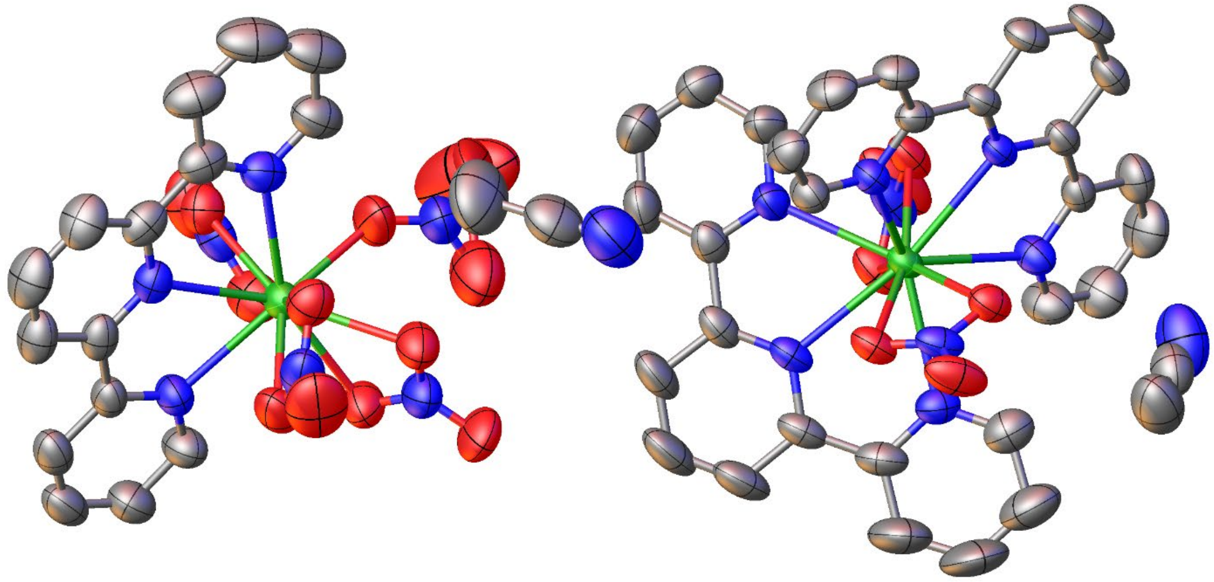

Figure S4. Crystal Structure of PmTerpy (CCDC# 2366328) shown at the 50% probability level. Hydrogens have been omitted for clarity.

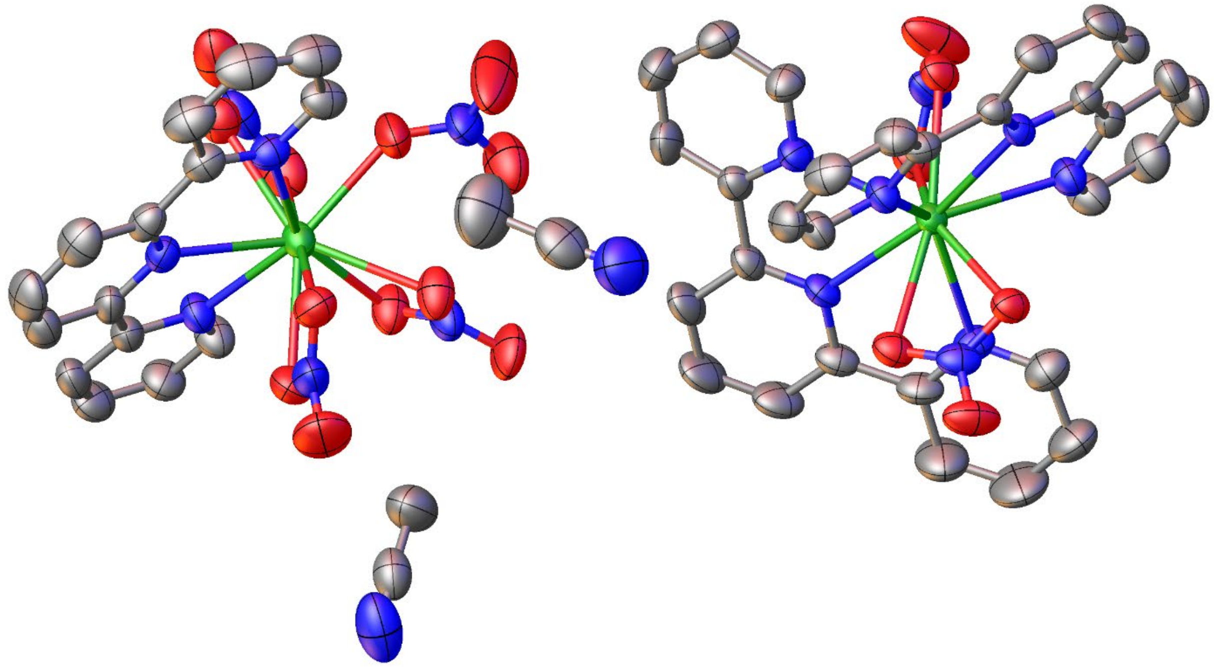

Figure S5. Crystal Structure of EuTerpy (CCDC# 2366327) shown at the 50% probability level. Hydrogens have been omitted for clarity.

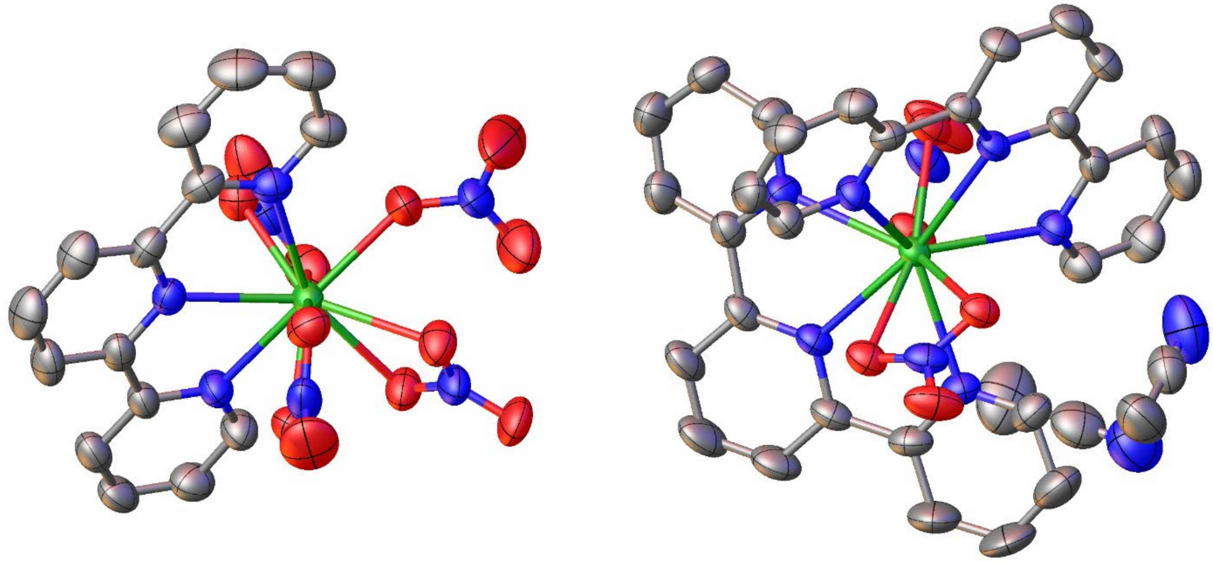

Figure S6. Crystal Structure of GdTerpy (CCDC# 2366324) shown at the 50% probability level. Hydrogens have been omitted for clarity.

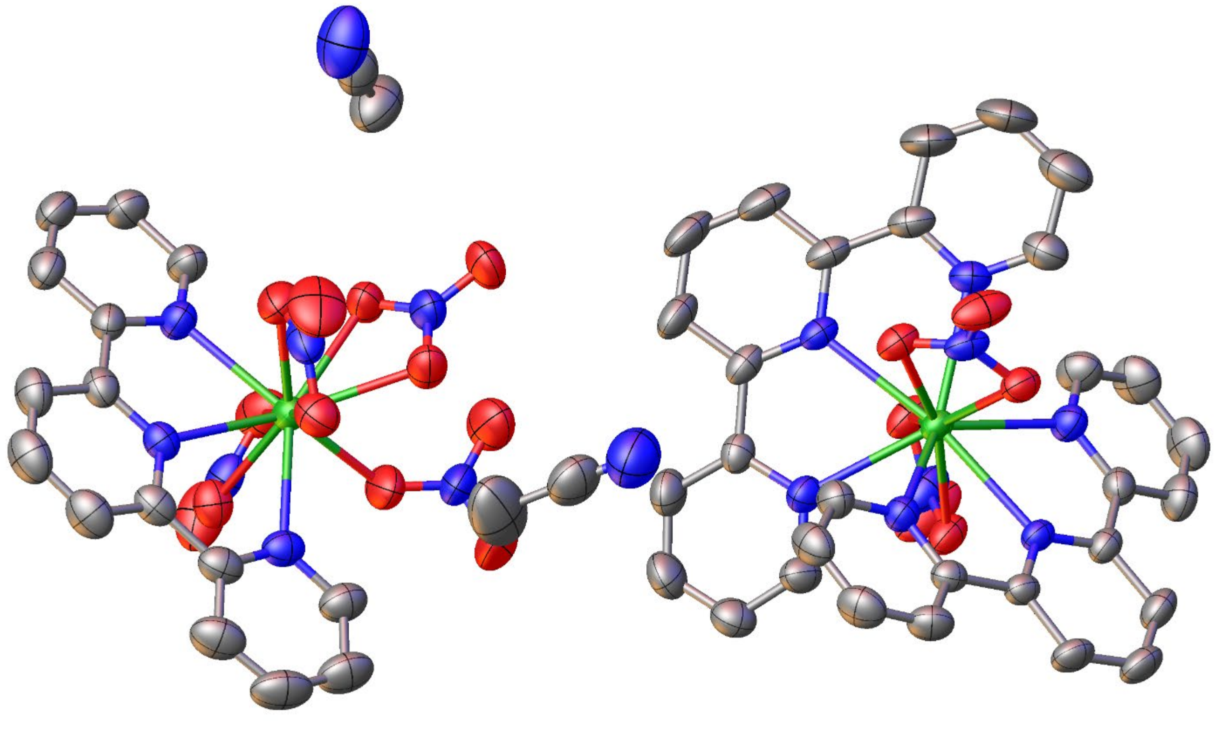

Figure S7. Crystal Structure of ErTerpy (CCDC# 2366323) shown at the 50% probability level. Hydrogens have been omitted for clarity.

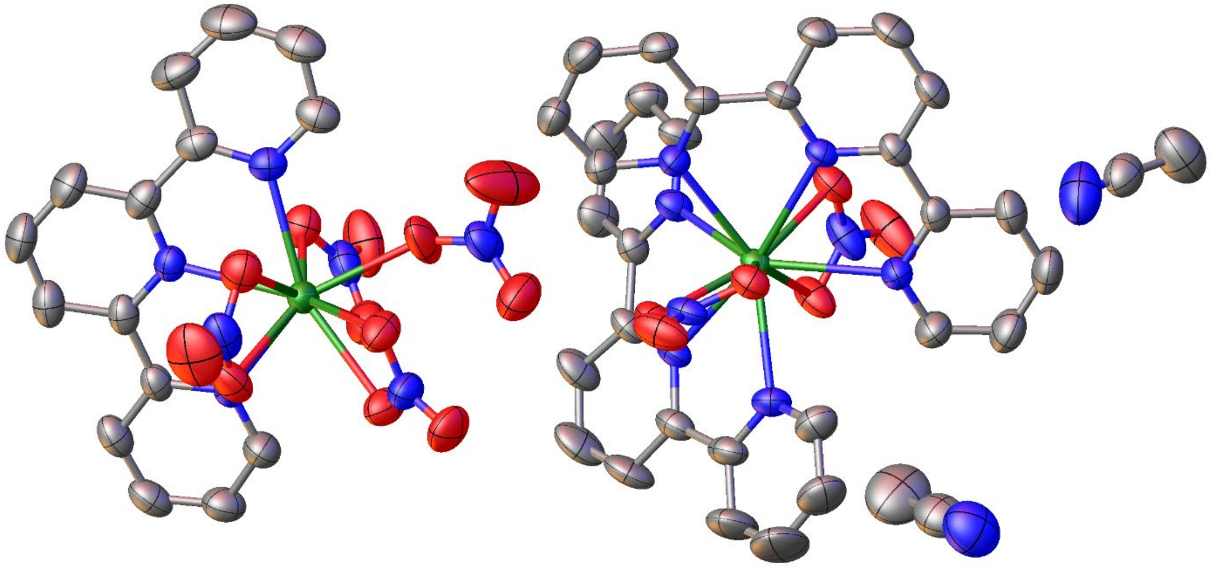

Figure S8. Crystal Structure of CmTerpy (CCDC# 2366326) shown at the 50% probability level. Hydrogens have been omitted for clarity.
